# High-Dimensional Medial Lobe Morphometry: An Automated MRI Biomarker for the New AD Diagnostic Criteria

**DOI:** 10.1155/2014/278096

**Published:** 2014-08-31

**Authors:** Simon Duchesne, Fernando Valdivia, Abderazzak Mouiha, Nicolas Robitaille

**Affiliations:** ^1^Départment de Radiologie, Faculté de Médecine, Université Laval, Quebec, QC, Canada G1V 0A6; ^2^Institut Universitaire de Santé Mentale de Québec, 2601 de la Canardiére/F-3582, Quebec, QC, Canada G1J 2G3

## Abstract

*Introduction*. Medial temporal lobe atrophy assessment via magnetic resonance imaging (MRI) has been proposed in recent criteria as an *in vivo* diagnostic biomarker of Alzheimer's disease (AD). However, practical application of these criteria in a clinical setting will require automated MRI analysis techniques. To this end, we wished to validate our automated, high-dimensional morphometry technique to the hypothetical prediction of future clinical status from baseline data in a cohort of subjects in a large, multicentric setting, compared to currently known clinical status for these subjects. *Materials and Methods*. The study group consisted of 214 controls, 371 mild cognitive impairment (147 having progressed to probable AD and 224 stable), and 181 probable AD from the Alzheimer's Disease Neuroimaging Initiative, with data acquired on 58 different 1.5 T scanners. We measured the sensitivity and specificity of our technique in a hierarchical fashion, first testing the effect of intensity standardization, then between different volumes of interest, and finally its generalizability for a large, multicentric cohort. *Results*. We obtained 73.2% prediction accuracy with 79.5% sensitivity for the prediction of MCI progression to clinically probable AD. The positive predictive value was 81.6% for MCI progressing on average within 1.5 (0.3 s.d.) year. *Conclusion*. With high accuracy, the technique's ability to identify discriminant medial temporal lobe atrophy has been demonstrated in a large, multicentric environment. It is suitable as an aid for clinical diagnostic of AD.

## 1. Introduction

### 1.1. Medial Temporal Lobe Atrophy as a Structural Biomarker of Alzheimer's Disease Progression

Early identification of patients most at risk of progression to dementia due to Alzheimer's disease (AD) remains a crucial clinical and research issue. To address this concern new criteria have been proposed to increase diagnostic certainty and better identify individuals in a prodromal state, mild cognitive impairment (MCI) due to AD [1–3].* In vivo *biomarkers of disease progression, both chemical and imaging, lie at the heart of these criteria.

The earliest AD-associated brain alterations, according to histopathological staging [4], occur in medial temporal lobe structures, in particular the hippocampus and entorhinal cortices; they have been reported in amnestic MCI subjects [5, 6]. The AD neurodegenerative cascade results in dendritic pruning, loss of synapses, and eventually neuronal death, resulting in cerebral atrophy of which structural magnetic resonance imaging (MRI) is able to measure. Thus, medial temporal atrophy (MTA) has been reported extensively on the continuum from MCI to AD [7, 8] and is a recognized imaging biomarker in the new criteria [1–3].

The most validated procedure to estimate MTA relies on expert manual outlining (i.e., segmentation) of individual or ensembles of structures on high resolution T1-weighted MRI, following an established set of anatomical landmarks [9]. While manual segmentation is accepted as the best available technique, it cannot be widely used within a large-scale clinical setting, as the investment in expertise and resources is prohibitively great. This type of application thus necessitates semiautomated or ideally completely automated image processing techniques, as a cost-efficient strategy.

### 1.2. Automated Techniques for MTA Assessment

There has been renewed enthusiasm recently over the performance of multi-atlas or template-based approaches for automated segmentation [10–16]. Over the last decadehowever a number of high-dimensional morphometry techniques have arisen that attempt to characterize potentially multimodal image information from a volume of interest larger than a single structure, generally encompassing the medial temporal lobe, and embedding machine learning principles to both characterize and discriminate subject populations [17]. There is increasing evidence that this approach will allow for more accurate determination of AD time course in a number of reports [18–27]. Other notable works include Davatzikos and colleagues with a diffeomorphing-based algorithm to extract high-dimensional patterns [28]; and Hua et al., who used tensor-based morphometry for similar purposes [29].

Our methodology is set within this context. It incorporates local estimates of both tissue composition and deformation within a specific volume of interest (VOI) centered on the medial temporal lobes [30], providing a structural index related to disease progression [31]. Such changes in tissue composition have been reported via voxel-based morphometry [32] and contrast studies [33], while volumetry [34] and tensor-based morphometry [29, 35] reports have shown pathology-related deformations in specific brain areas. By incorporating both image features, we are able to capture different properties of the advancing pathological process and predict future clinical status for an individual subject. We have applied this methodology in earlier work to the discrimination of probable AD from age-matched healthy controls [30] as well as the prediction of amnestic MCI progression to clinically probable AD [36], albeit within a single-center setting.

### 1.3. Bridging the Gap towards Clinical Use

Clinical application of any one of these automated methodologies require that techniques maintain the same level of performance in a multicentric setting, where large interscanner variations become inevitable due to MRI physics [37], even though systematic errors (such as different acquisition protocols) are controlled. These random effects will distort image intensities, which in turn will influence image processing and, eventually, classification performance. Not all techniques proposed in the literature have been subjected to this kind of sensitivity analysis.

An ideal dataset for this purpose is the Alzheimer's Disease Neuroimaging Initiative (ADNI) study [38]. For the first phase of ADNI, a total of 822 subjects (229 normal controls (CTRL), 405 individuals with MCI, and 188 subjects with mild AD) were recruited in 58 sites throughout the United States and Canada for longitudinal followup. ADNI was successful in coordinating and implementing a routine imaging protocol at all sites with stringent quality control [39, 40], thereby ensuring that all scans were similarly acquired, reducing systematic errors. Cuingnet et al. conducted a comparative study of ten machine learning techniques using ADNI data [18] in which many parameters were controlled. Such reports are useful for benchmarking and serve to improve system's performance and robustness.

### 1.4. Study Objectives

Our general objective is to assess the accuracy of our automated high-dimensional morphometry technique to the hypothetical prediction of future clinical status from MRI when examining previously acquired data in a cohort of MCI subjects from the large, multicentric ADNI dataset, compared to the currently known clinical status for these subjects, under various conditions.

Specifically, we will want to test the following hypotheses, which would need to hold true for any methodology:

- that intensity standardization and tissue classification improve the system's robustness and hence performance, in a multicentric setting;
- that a medial temporal lobe VOI is the best for the differentiation of CTRL from either probable AD or MCI progressing to probable AD, as opposed to whole-brain VOIs [43];
- that the methodology remains highly accurate even with large, ostensibly heterogeneous datasets.

Proving or disproving these hypotheses would constitute significant contributions that could be further employed in other, similar research endeavors.

## 2. Methods

### 2.1. Ethics

Each participant from the ADNI cohort was formally evaluated using eligibility criteria that are described in detail elsewhere (http://www.adni-info.org/). The institutional review boards of all participating institutions approved the procedures for this study. Written informed consent was obtained from all participants or surrogates. More information about the ADNI investigators is given in Acknowledgment.

### 2.2. Study Design

This is a retrospective analysis of data from a nonrandomized, natural history nontreatment study.

### 2.3. Subjects' Data

Inclusion criteria to the ADNI (Data used in the preparation of this article were obtained from the Alzheimer's Disease Neuroimaging Initiative (ADNI) database (http://www.loni.usc.edu/ADNI/). The ADNI was launched in 2003 by the National Institute on Aging (NIA), the National Institute of Biomedical Imaging and Bioengineering (NIBIB), the Food and Drug Administration (FDA), private pharmaceutical companies, and nonprofit organizations, as a $60 million, 5-year public-private partnership. The primary goal of ADNI has been to test whether serial magnetic resonance imaging (MRI), positron emission tomography (PET), other biological markers, and clinical and neuropsychological assessment can be combined to measure the progression of mild cognitive impairment (MCI) and early Alzheimer's disease (AD). Determination of sensitive and specific markers of very early AD progression is intended to aid researchers and clinicians to develop new treatments and monitor their effectiveness, as well as lessening the time and cost of clinical trials. The principle investigator of this initiative is Michael W. Weiner, M.D., VA Medical Center and University of California, San Francisco. ADNI is the result of efforts of many coinvestigators from a broad range of academic institutions and private corporations, and subjects have been recruited from over 50 sites across the U.S. and Canada. The initial goal of ADNI was to recruit 800 adults, ages 55 to 90, to participate in the research—approximately 200 cognitively normal older individuals to be followed for 3 years, 400 people with MCI to be followed for 3 years, and 200 people with early AD to be followed for 2 years. For up-to-date information see http://www.adni-info.org/.) study were as follows:

- CTRL: MMSE scores [44] between 24–30 (inclusive), a CDR [45] of 0, non-depressed, non-MCI, and nondemented. The age range of normal subjects was roughly matched to that of MCI and mild AD subjects;
- MCI subjects: MMSE scores between 24 and 30 (inclusive), a memory complaint, have objective memory loss measured by education adjusted scores on Wechsler Memory Scale Logical Memory II [46], a CDR of 0.5, absence of significant levels of impairment in other cognitive domains, essentially preserved activities of daily living, and an absence of dementia;
- Mild AD: MMSE scores between 20 and 26 (inclusive), CDR of 0.5 or 1.0, and meets NINCDS/ADRDA criteria for probable AD [47].

**Figure 1:**
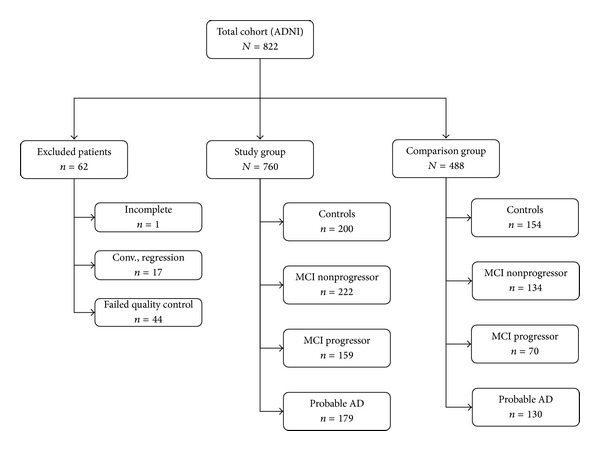
Cohort flow diagram.

From the complete ADNI dataset of 822 subjects at baseline, we selected individuals for the* Study Group *that met the following criteria (*cf*.  Figure 1): (a) valid entry images; (b) processed images that passed* automated *quality control; (c) long-term clinical assessment; and (d) no conversion (CTRL) or regression (Mild AD, MCI) in terms of final diagnostic.

*In fine*, the* Study Group* was composed of 200 CTRL, 179 patients with Mild AD, and 381 MCI subjects (*cf*.  Table 5 for a list of quality-control exclusions). Within the MCI population, 159 MCI progressed to clinically probable or possible AD (MCI-P) at an average followup of 1.5 years (SD: 0.3 years; range 0.1–3.5 years), while 222 remained stable (MCI-NP) within an average followup of 2.2 years (SD: 1.0 years; range 0.0–4.1 years).

In order to benchmark our technique with the literature, we selected 488 subjects used in the Cuingnet study [18] and formed the* Comparison Group*, in effect a subset of the larger* Study Group*. The difference between ours and the Cuingnet listing are quality control rejections from our study. By using similar groups, we allow external validation of our results with the literature.

### 2.4. MRI Acquisitions

MRI data for all* Study Group* subjects were acquired on 58 different 1.5T scanners (GE Medical Systems; Siemens Healthcare; Philips Healthcare) using a 3D T1-weighted MP-RAGE protocol or its equivalent [40].

### 2.5. MRI Preprocessing

We processed all raw MRI acquisitions in an identical fashion: (a) DICOM to MINC (http://www.bic.mni.mcgill.ca/) conversion; (b) raw scanner intensity inhomogeneity correction [48]; (c) noise removal based on a 3D optimized blockwise version of the nonlocal-means filter [49]; (d) linear scaling of grey level intensities to match the mean level of the reference image; (e) global registration (12 degrees of freedom) to the reference image space [50], maximizing the mutual information between the two volumes [51]; (f) resampling to a 1 mm^3^ isotropic grid; (g) intensity standardization and tissue classification (see Section 2.6) to the reference image intensity histogram; (h) tissue classification into cerebrospinal fluid, grey matter (GM), and white matter components; (i) nonlinear image registration [52] to assess differences between any given subject and the reference image; and (j) computation of the determinant of the Jacobian of the dense deformation fields mapping the subject's volume to the reference image. The determinant represents a biologically meaningful quantity; in this case, an estimate of local brain tissue volume difference between the individual and the reference volume. When the difference is near zero, there is no local difference in volume between subject and reference images. However, if the determinant is positive, the volume is larger, whereas when negative, the volume is smaller when compared to the reference after the deformation. It would be possible to integrate the resulting values to obtain volumetric estimates, which it not our intent at this point.

The reference image was an unbiased standard magnetic resonance imaging template brain volume for a young adult population, created using data from the ICBM project [53].

We did not perform distortion correction, nor selected images corrected for distortion from the ADNI distribution website. We assessed—albeit visually—that our fully affine linear registration, centered on the medial temporal lobe, was sufficient to remove most of the effects.

### 2.6. Processing Variables

#### 2.6.1. Intensity Standardization and Tissue Classification

The problem of multicentric acquisitions is to ensure that similar intensities will have analogous tissue meaning in the images across scanners. In this study we tested three intensity features: (i) T1-weighted intensities, scaled to match the mean level of the reference image (*cf*.  Section 2.5 (d)); (ii) T1-weighted intensities after undergoing a standardization process [54]; and (iii) grey matter (GM) probability maps, obtained via a tissue classification algorithm performed on the scaled intensity images [55].

**Figure 2:**
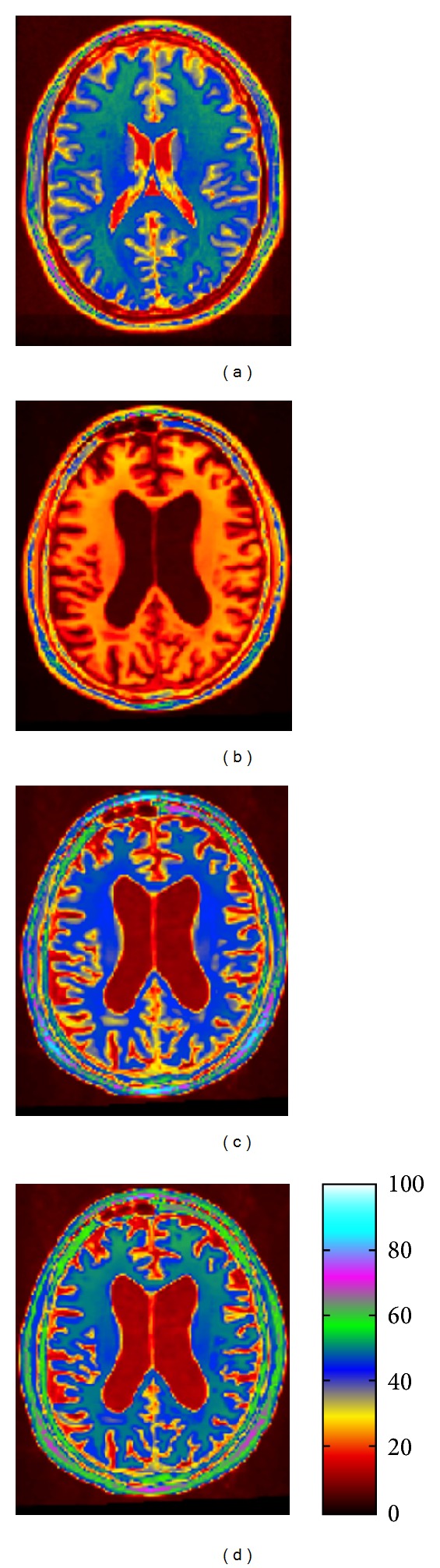
Intensity standardization example for an ADNI subject. From left to right: (a) reference image; (b) original image; (c) standardized image using the Nyul et al. histogram-matching technique [41]; and (d) standardized image using our tissue derived, spatially constrained intensity matching technique [42]. The color map was chosen to increase contrast.

The intensity standardization technique makes use of available reference image tissue masks (background, grey matter and white matter). After global linear registration of the subject's image to the reference, a piecewise linear mapping function is computed based on the intensity correspondences obtained for each tissue, thereby implicitly binding histogram matching to tissue correspondence (rather than only matching histograms, as is the case in a number of different techniques, e.g., [41]). The following steps are performed for each tissue: (1) mask both subject and reference images; (2) compute and smooth the subject-reference joint intensity histogram; (3) find joint tissue maxima; (4) determine the intensity mapping function by interpolating linearly between maximum tissue positions; and (5) apply the mapping to the original linear-registered image (see Figure 2).

The GM probability maps were obtained by feeding intensity images to a neural network classifier [56], which provided fuzzy probability maps for each tissue class, from which we retained only the GM probability.

We visually inspected and compared all standardized images and GM probability maps for quality control.

#### 2.6.2. Volumes of Interest

**Figure 3:**
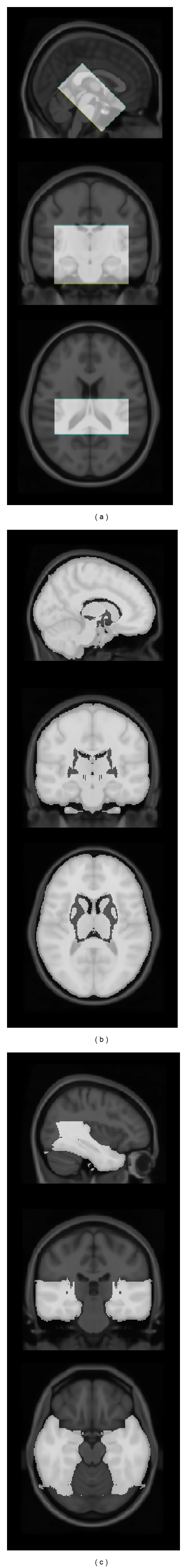
Overview of (a) medial temporal lobe volume of interest; (b) whole brain mask; and (c) temporal lobe volume of interest.

Following the conclusions of Pelaez-Coca et al. [43], we tested two additional VOIs in addition to the cubic-shaped MTL volume from our previous study [30]. The anatomical VOIencompassed all of the temporal lobe as well as the ventricles, as defined on segmentation probability maps from the reference image [53]. The global VOI encompassed the whole cerebrum, as defined via a mask on the template reference image. All three volumes are shown in Figure 3.

#### 2.6.3. Study Groups

All of the previous tests were done using the complete* Study Group*, in effect testing for generalizability. Further, to benchmark our technique with the literature, we used the* Comparison Group*, in effect the same subjects used in the Cuingnet study [18] (bar quality control exceptions). By using similar groups, we allow external validation of our results with the literature.

### 2.7. Classification

The classification method we employed is summarized below. It builds on the previous methodology described elsewhere [30].

First, the* Study Group *was randomly split into* Training *and* Testing *groups.

Next, we generated from the* Training Group *a representative feature space by performing principal component analysis of (i) image intensities within the VOI as a proxy of local tissue composition and (ii) image determinants as a proxy of local tissue differences. We then expressed the* Training Group *data as coordinates in the new principal components space, and we assessed normality of the univariate distributions of coordinates along any principal component via Shapiro-Wilk statistics and rejected nonnormal distributions.

We used support vector machines with a linear kernel to select the discriminatory variables from the projected data forming the best discriminating function in the* Training Group *for the classification task at hand (e.g., CTRL* versus* Probable AD; CTRL* versus* MCI-P; MCI-P versus MCI-NP). To complete the analysis, we projected the* Testing Group *in the same principal components space, and used the discrimination function to obtain independent assessment of the system's accuracy. To ensure we did not have a particular bias related to random group assignments in the* Study Group*, we repeated the random assignment process ten times.

We performed modeling, statistical, and classification analyses using MATLAB (The MathWorks, Natick, MA).

### 2.8. Reference Standard

The reference standard for classification consisted of the latest, longitudinal clinical assessment available through ADNI.

### 2.9. Experimental Design

We tested our three hypotheses in a hierarchical fashion, namely, as follows.

- Testing first for robustness, using either the T1-weighted intensities (Step 2.5(d)), standardized T1-weighted intensities (Step  2.5(g)) or the GM probability maps following intensity standardization (Step  2.5(h)), in the* Study Group *and within the cubic-shaped VOI.
- Testing next for spatial sensitivity, using either the cubic-shaped,* Anatomical *or* Global *VOIs, in the* Study Group* and with the best intensity feature obtained in the previous step.
- Testing finally for comparison, using both the ADNI* Study Group *and the Cuingnet* Comparison Group*, in the best VOI and with the best intensity feature obtained from previous steps.

### 2.10. Statistical Analysis

The final reported results are averaged over all trials for accuracy, sensitivity, and specificity. We further employed McNemar's test using exact binomial probability calculations to assess the significance of the difference between the two correlated proportions of the truth table (clinical assessment versus MRI assessment).

### 2.11. Role of the Funding Sources

The funders had no role in study design, data collection and analysis, decision to publish, or preparation of the manuscript.

## 3. Results

### 3.1. Subjects

The first phase of the ADNI study was closed for recruitment on October 23, 2008.

After removing subjects for which incomplete data existed at follo-up or that failed anyone of the image processing steps (see Table 5), there were 760 subjects in the* Study Group* (see Figure 1) and 488 subjects in the* Comparison Group*.

Demographic information (age, sex) for each diagnostic subgroup are reported in Table 1.

### 3.2. Robustness Testing

We used principal components analysis to reduce the dimensionality of subjects' data to generate two linear variation models of image intensities and local volume differences as proxies of tissue composition and deformations. For both models, we retained features that explained 68% of the variance of the input data.

The best results were obtained with the GM probability maps (see Table 2). In terms of accuracy the discrimination of CTRL from probable AD in the* Study Group* was 77.9% (189/243), sensitivity 76.3% (90/118), and specificity 79.2% (99/125). By using McNemar's Test (chi-square statistics with 1 ddl: 0.0741; *P* value = 0.7855), the difference is not significant. Results for the discrimination of CTRL from MCI-P (Table 3) were 72.2% (205/284), sensitivity 79.2% (126/159), and specificity 63.4% (79/125). Likewise, the MRI-clinical test results are not statistically different (McNemar test: chi-square statistics with ddl = 1 : 2.1392; *P* value = 0.1436, the difference is not significant). Finally, results for the discrimination of MCI-P from MCI-NP (Table 4) were 62.2% (237/381), sensitivity 34.6% (55/159), and specificity 82.0% (182/222). For the MRI-clinical test results are statistically different (McNemar test, chi-square statistics with ddl = 1 : 28.444, *P* value < 0.0001).

### 3.3. Spatial Sensitivity Testing

To test the influence of VOI, we retrained the system using GM probability maps and determinant information in each of the three VOIs. In each case we retained features that explained 68% of the variance of the input data.

The best results in terms of accuracy for discrimination were obtained using the same cubic-shaped VOI as in Section 3.2 and hence provided similar results for CTRL versus AD, CTRL versus MCI-P, and MCI-P versus MCI-NP.

### 3.4. Generalizability Testing

All of the previous results were obtained with the more inclusive* Study Group* and averaged over 10-fold. For comparison and benchmarking purposes, we used the best technique from previous test and applied it to the Cuingnet* Comparison *Group, which was split only once in the same* Training/Testing *sets as their original article. Results show accuracy for discrimination of CTRL from probable AD of 78.7% (107/136), sensitivity 72.5% (50/69), and specificity 85.1% (57/67) (Table 2). These results are not significant, that is, the McNemar statistical test rejects sthe null hypothesis (chi-square statistics with ddl = 1 : 2.7931; *P* value = 0.0947). Results for the discrimination of CTRL from MCI-P were 59.4% (60/101), sensitivity 82.4% (28/34), and specificity 47.8% (32/67) (Table 3). McNemar test is strongly indicative of congruence (chi-square statistics with ddl = 1 : 20.5122; *P* value < 0.0001). Finally, discrimination of MCI-P from MCI-NP were 66.0% (64/97), sensitivity 2.94% (1/34), and specificity 100% (63/63) (Table 4). McNemar test is also strongly indicative of congruence (chi-square statistics with ddl = 1 : 33.00; *P* value < 0.0001).

## 4. Discussion

### 4.1. Clinical Applicability

We wished to assess the ability of our T1-weighted MRI classification technique to the retrospective, cross-sectional prediction of future clinical status in a cohort of subjects within the large, multi-centric ADNI cohort, under various conditions.

Our technique achieved a high level of performance for the discrimination of probable AD from CTRL, achieving 79% accuracy on a comparative, benchmarked cohort, and 78% in a nearly twice-larger dataset. These results are statistically comparable to the clinical diagnostic (as per McNemar's test) and thus support the use of machine-learning techniques such as ours as biomarkers of medial temporal lobe atrophy within expanded criteria for the diagnostic of probable AD, such as proposed by McKhann et al. [2]. The technique also reached a global accuracy of 62% for progression of MCI to probable AD within 1.5 years on average after baseline, also congruent with a clinical diagnostic. These results indicate that specific spatially covarying intensity and local volume difference patterns, representative of tissue composition and deformation at that instant in time hold discriminatory information related to future clinical status in MCI. As for the discrimination of MCI-P from MCI-NP, further improvements are required if MRI alone is to be used. It remains that the most probable course of action is to pair up MRI information with clinical/cognitive testing.

**Figure 4:**
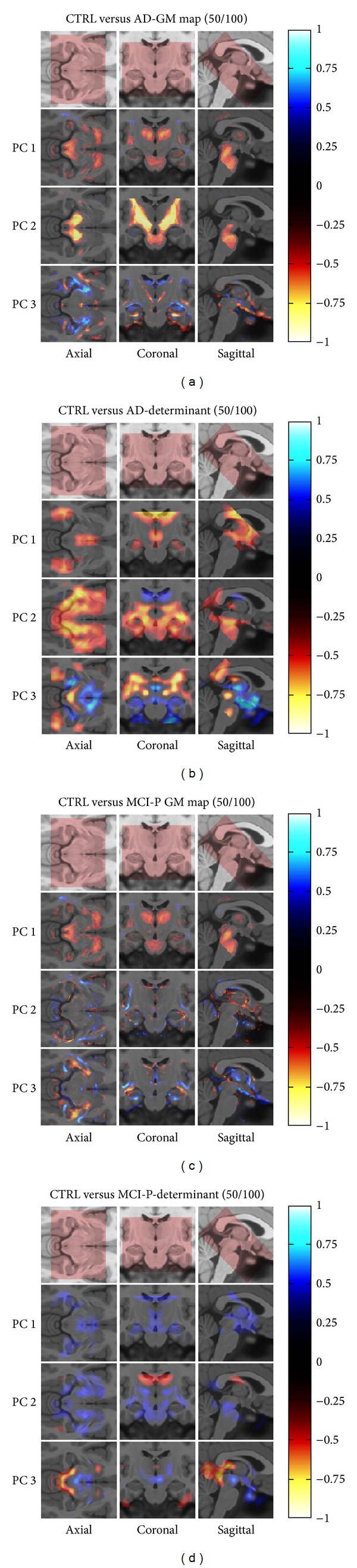
Significant structural differences within the medial temporal lobe related to the discrimination task between (a, b) CTRL versus probable AD and (c, d) CTRL versus MCI-P. Left images represent grey matter concentration differences, while right images represent deformation differences. For each map, we present the covarying voxels associated with the top three eigenvectors in each discriminating function, color-coded with respect to their negative or positive distance from the center and normalized to the maximum absolute value in the VOI.

We explored in Figure 4 the spatial distribution of discriminating information for GM or determinant differences between CTRL versus AD (Figures 4(a) and 4(b)) and CTRL versus MCI-P (Figures 4(c) and 4(d)). The results show an expected distribution of atrophy around the hippocampal and ventricular areas, which follow the expected atrophy distribution demonstrated in prior neuropathological studies (e.g., Braak stages I–VI) [57]. Thus, these results lead us to conclude that the automated technique is able to track discriminant medial temporal lobe atrophy characteristics related to AD, and thus serve as an aid for said diagnostic in a clinical setting. As well, this can be thought of as a biomarker of interest for neurodegeneration in MCI due to AD, as recommended by Albert et al. [3].

A number of previous reports have explored the topic of MR-based classification and prediction [18, 19, 21, 22, 24–26]. Fewer authors have explored multimodal (e.g. MRI and FDG-PET [20, 27]; MRI and SPECT [23]) or multifactorial (e.g., MRI and CSF [58, 59]). Some of the latter report higher discriminatory abilities when using multimodal information. However, further evidence is required for those studies, as on the one hand cohort sizes remain small (especially for multi-modal studies), and on the other, the acquisition process becomes clinically expensive and demanding for the patients.

Our results are in line with this previous literature but are best compared to studies using similar datasets. Therefore, the benchmarking study by Cuingnet et al. [18] is especially valuable. It should be pointed that their results show only four techniques out of 10 scoring accuracies above chance at the MCI-P versus NP discrimination task. The performance of our technique thus becomes positively validated.

### 4.2. Limitations

A substantial limitation of this study and, so far for all studies based on the ADNI dataset, remains the lack of histopathological confirmation for AD cases and MCI progression. Even though the longitudinal followup duration was substantial, our results do not equate perfectly with predicting AD, as the clinical assessment is not inherently 100% accurate. The length of followup is also expected to bias the results as more MCI subjects are expected to progress to clinically probable AD.

It should also be noted that we used a reference image created from the ICBM project. The choice of a reference image has been shown for other techniques to have a significant outcome on final results. We have tested this hypothesis early on (results not shown) using various templates, including age-related templates and did not uncover appreciable differences for the discrimination tasks that we explored. However, this cannot be construed as a general rule, since the choice of template may influence other discrimination tasks and should therefore be verified each time.

Finally, albeit the ADNI dataset is large, we must use machine-learning approaches to optimize training/testing, and *k*-fold validation is one such well-known method. The unavoidable downside to this approach is that slightly different information is collected for each fold. Thus, in a strict statistical sense, it is likely that the results are an overestimation of the classification rate on generalized data.

Our results indicate that our completely automated technique is able to extract critical individualized diagnostic information from standardized MRI acquisitions, obtainable in a clinical setting.

## Figures and Tables

**Table 1:** Demographics.

Group	CRTL (*n* = 200)	AD (*n* = 179)	MCI-NP (*n* = 222)	MCI-P (*n* = 159)	*P*
Age (years)	75.8	75.1	74.9	74.8	0.4807
Sex (%F)	48%	48%	34%	40%	0.0069

A one-way ANOVA is used to compare group ages and a chi-square test to compare sex.

**Table 2:** Discrimination of controls versus probable AD.

Data set	Data type	VOI	Correct Rate	Sn	Sp	Sn + Sp
Intensity standardisation testing						
Study	Original intensity + determinant	MTL	0.741	0.705	0.776	
Study	STI intensity + determinant	MTL	0.744	0.732	0.758	
Study	GM + determinant	MTL	0.779	0.763	0.792	

Volume of interest testing						
Study	GM + determinant	MTL	0.779	0.763	0.792	1.652
Study	GM + determinant	Segmented	0.778	0.739	0.815	
Study	GM + determinant	Brain	0.691	0.660	0.718	

Large-scale testing						
Study	GM + determinant	MTL	0.779	0.763	0.792	
Comparison	GM + determinant	MTL	0.787	0.725	0.851	

STI: intensity standardisation technique; GM: grey matter; VOI: volume of interest; MTL: medial temporal lobe volume; Sn: sensitivity; Sp: specificity.

**Table 3:** Discrimination controls versus MCI progressors.

Data set	Data type	VOI	Correct rate	Sn	Sp	Sn + Sp
Intensity standardisation testing						
Study	Original intensity + Determinant	MTL	0.700	0.742	0.649	
Study	STI intensity + determinant	MTL	0.721	0.780	0.623	
Study	GM + determinant	MTL	0.722	0.792	0.634	

Volume of interest testing						
Study	GM + determinant	MTL	0.722	0.792	0.634	
Study	GM + determinant	Segmented	0.708	0.730	0.681	
Study	GM + determinant	Brain	0.683	0.785	0.550	

Large-scale testing						
Study	GM + determinant	MTL	0.722	0.792	0.634	
Comparison	GM + determinant	MTL	0.594	0.824	0.478	

**Table 4:** Discrimination of MCI progressors versus nonprogressors.

Data set	Data type	VOI	Correct rate	Sn	Sp	Sn + Sp
Intensity standardisation testing						
Study	Original intensity + determinant	MTL	0.606	0.278	0.843	
Study	STI intensity + Determinant	MTL	0.635	0.372	0.824	
Study	GM + determinant	MTL	0.622	0.346	0.820	

Volume of interest testing						
Study	GM + determinant	MTL	0.622	0.346	0.820	
Study	GM + determinant	Segmented	0.612	0.340	0.794	
Study	GM + determinant	Brain	0.572	0.145	0.878	

Large-scale testing						
Study	GM + determinant	MTL	0.622	0.346	0.820	
Comparison	GM + determinant	MTL	0.660	0.029	1.000	1.029

**Table 5:** Quality control data for the ADNI cohort. Subjects included in this table have been excluded for analysis on the basis of (A) missing, badly formatted or wrong acquisition sequence of input images; (B) poor contrast/signal-to-noise ratio; (C) failure of automated processing for the pipeline described in this paper. Note that other image processing pipelines may/may not succeed/fail for identical subjects.

57	77	161	177
194	259	273	282
311	312	325	326
406	431	433	446
575	598	602	618
621	629	633	679
686	747	850	855
860	928	931	991
1073	1131	1188	1205
1261	1331	1339	1343
1391	1407	1412	1419

## Abbreviations

AD: Alzheimer's disease
ADNI: Alzheimer's Disease Neuroimaging Initiative
aMCI: Amnestic MCI
CTRL: Control subjects
GM: Grey matter
MCI: Mild cognitive impairment
MRI: Magnetic resonance imaging
MTA: Medial temporal lobe atrophy
MCI-NP: MCI nonprogressors
MCI-P: MCI progressors
NPV: Negative predictive value
PPV: Positive predictive value
VOI: Volume of interest.
